# Unraveling athletic performance: Transcriptomics and external load monitoring in handball competition

**DOI:** 10.1371/journal.pone.0299556

**Published:** 2024-03-11

**Authors:** Pol Ezquerra Condeminas, Milos Mallol, Roger Font, Victor Tremps, Jose Antonio Gutiérrez, Gil Rodas, Alexandre Perera Lluna, José Manuel Soria

**Affiliations:** 1 Department of Innovation and Development Area, Beyond You (Exheus S.L.), Barcelona, Spain; 2 b2slab, Universitat Politècnica de Barcelona (UPC), Barcelona, Spain; 3 Performance Department of Football Club Barcelona, Barcelona, Spain; 4 Barça Innovation Hub of Football Club Barcelona, Barcelona, Spain; 5 Department of Health Sciences, Research group in Technology Applied to High Performance and Health (TAARS), Tecnocampus, Pompeu Fabra University, Mataró, Spain; 6 National Institut of Physical Education of Catalonia (INEFC), GRCE Research Group, Barcelona, Spain; 7 Department of Sports Sciences, Ramon Llull University, FPCEE Blanquerna, Barcelona, Spain; 8 Medical Department of Football Club Barcelona (FIFA Medical Centre of Excellence), Barcelona, Spain; 9 Networking Biomedical Research Centre in the subject area of Bioengineering, Biomaterials and Nanomedicine, Madrid, Spain; 10 Institut de Recerca Sant Joan de Deu, Barcelona, Spain; 11 Unit of Genomics of Complex Disease, Research Institute of Sant Pau Hospital (IIB Sant Pau), Barcelona, Spain; 12 Instituto de Salud Carlos III, Centre for Biomedical Network Research on Rare Diseases (CIBERER), Madrid, Spain; Instituto Politecnico de Viana do Castelo, PORTUGAL

## Abstract

**Objective:**

This study aims to comprehend the impact of handball practice on sub-elite athletes by investigating transcriptomic changes that occur during a match. The primary focus encompasses a dual objective: firstly, to identify and characterize these transcriptomic alterations, and secondly, to establish correlations between internal factors (gene expression), and external loads measured through Electronic Performance and Tracking Systems (EPTS variables). Ultimately, this comprehensive analysis seeks to evaluate both acute and chronic responses to exercise within the context of handball training.

**Methods:**

The study included sixteen elite male athletes from the FC Barcelona handball second team. Blood samples were extracted at three different time points: before the match at baseline levels (T1), immediately upon completion (T2), and 24 hours after completion (T3). Differential gene expression, Gene Ontology Term and Kyoto Encyclopedia of Genes and Genomes pathway enrichment analyses were conducted in two comparisons: Comparison 1 (T1 vs T2) and Comparison 2 (T1 vs T3). Further, the correlation between gene expression levels and training variables (external load) was conducted.

**Results:**

In T1 vs T2, 3717 of the 14632 genes detected were differentially expressed (adjusted p-value < 0.05), and enrichment of terms related to the immune system, mitochondria, and metabolic processes was found. Further, significant linear correlations were obtained between High-Speed running (HSR) and high-intensity variables such as acceleration ACC and deceleration DEC values with amino acids, and inflammatory and oxidative environment-related pathways, both in chronic and acute response.

**Conclusions:**

This research highlights the effects of external workload on elite athletes during a handball match and throughout the season. The study identifies deregulation in the immune system, mitochondrial functions, and various metabolic pathways during the match. Additionally, it establishes correlations between the external load and pathways associated with amino acids, inflammation, oxidative environment, and regulation. These findings offer insights into the immediate and chronic responses of athletes to physical effort.

## Introduction

Athletes undergo rigorous and repetitive training regimens aimed at enhancing their physical capacities, including muscular power, endurance, and speed, while also striving to shorten recovery periods. Controlling both training and competition loads is crucial for athletes to perform at their peak while minimizing the risk of injury [1–5]. However, the design of training programs to optimize individual performance is not always easy and even more so in a sports team because of the existing intra- and inter-variability between athletes in their levels of adaptation to training loads [6].

Training load can be described as internal or external. External loads are objective measures of the work done by athletes, such as a sprint, running distance covered in a match, or the number of accelerations and decelerations that can be measured using electronic performance tracking systems (EPTS) [1,2,3,7]. The needs of handball players in official competitions have been examined concerning their specific external load requirements and according to their playing position [7–9]. Pivots are those players that cover the least distance (3149 ± 639 m), and wingers are those players that accumulate the most distance traveled at high intensity (1229 ± 129.4 m) and sprint [7,10].

On the other hand, internal load is defined as the athlete’s physiological responses to the imposed external load during training or competition [11]. Measuring internal load is more challenging because it involves the evaluation of both psychological (e.g., rating of perceived exertion [RPE]) and physiological aspects (e.g., heart rate [HR], lactate concentration in blood or oxygen consumption) [12].

Different investigations have focused on observing the effects of internal loads during handball competition [13–15]. Significant differences have been found depending on the playing position at the HR level [13,14]. For example, a previous study showed that back players and pivots had the highest mean HR values and total playing time at intensities of >80% HRmax [15]. These differences at the external load level add further disparity in the response to training, which indicates that individualization of work is necessary [16,17].

External and internal loads are complementary, as they represent distinct aspects of adaptation; conjoint analysis can offer information on process adaptation that cannot be achieved from a separate analysis of each type of load [18]. However, the association between external and internal loads is not straightforward because physiologically, the response to exercise depends on several factors, such as genetics, previous training loads, and level of physical condition [19]. In this sense, previous studies have focused on genomics [20,21] and metabolomics [22]. However, while specific gene expression responses have been previously studied in endurance exercise, the whole genomic transcriptomic analysis (expression of the entire genome) has not been fully characterized. [23].

The impact of external and internal loads can be measured in terms of their acute and chronic effects on the body. Chronic adaptations refer to changes that occur after 6–12 weeks of consistent exercise, while acute responses refer to the immediate or short-term changes in response to exercise that the body makes to meet the additional energy demands when transitioning from rest to exercise. It is important to note that acute and chronic exercise effects cannot be considered in isolation [24].

Therefore, the main goals of this study were to recognize transcriptomic changes occurring throughout a handball match in elite athletes and to determine the correlation between internal (gene expression) and external (variables) loads to measure acute and chronic responses to exercise.

## Methods and materials

### Participants

The total number of study participants was sixteen, but this varied depending on the specific analysis. Specifically, for the Differential Expression analysis, sixteen male handball players from FC Barcelona were involved. However, only eleven players were considered for the correlation analysis between external and internal load due to the availability of external load records. Among these participants, five lacked external load data, which included two goalkeepers, while the remaining players had incomplete external load variable records.

All of these athletes shared the commonality of belonging to the same team, where both their playing and training conditions were meticulously controlled, because they had the same personal trainer and nutritionist team. Additionally, each player adhered to an identical nutritional plan, recovery strategy, and physical preparation regimen, creating a controlled environment essential for the forthcoming rigorous analysis.

The weight and height averaged 82±7.79 kg and 188±11.2 cm, respectively. In contrast, the average age was 20.31±2.60 years.

The final variable that distinguished each player was their respective playing position during the match. It is, however, crucial to maintain control over these positions, as they can directly influence the external load recorded by their devices. This aspect is particularly advantageous for our study, as it introduces variability in the external load experienced by each player, enabling us to explore the differences in internal load resulting from variations in external load (Table 1).

**Table 1:** Fundamental metadata information about the players involved in this analysis.

HEIGHT (CM)	WEIGHT (KG)	PLAYER POSITION	AGE
**188**	80	Center Back / Back	18
**181**	72.1	Goalkeeper	18
**175**	72.2	Center Back	18
**194**	85	Winger	18
**184**	80	Center Back	20
**175**	79	Back	19
**190.5**	88	Pivot	19
**188**	82	Right Wing	22
**195**	101	Left Wing	21
**197**	95.6	Defensive Specialist	31
**194**	79	Back	20
**193**	86	Left Wing	20
**175**	75	Right Wing	19
**197**	102	Pivot	20
**186**	98	Goalkeeper	19
**193**	105	Right Wing / Center Back	29

### Study design

This was a prospective observational study. Transcriptomic information was obtained from whole blood samples at three different time points following a single handball match: T1, T2, and T3. T1 samples were extracted at the baseline level (just before starting it), T2 samples were extracted immediately after finishing the match, and T3 samples were collected 24 h after finishing it. On the other hand, the external load was inferred using the following variables: the relative and absolute high-speed running (HSR), the acceleration, the deceleration, the minutes, and the total distance covered during a whole season and the average RPE, which measures the intensity of the exercise.

To assess the internal load, whole blood samples were utilized, which were subjected to a series of processes, including RNA extraction, sequencing, and quantification. This comprehensive approach allowed us to accurately determine the gene expression levels for each identified gene, providing valuable insights into the physiological responses of the athletes. On the other hand, the evaluation of the external load was conducted using Electronic Performance and Tracking Systems (EPTS). Furthermore, both acute and chronic values were considered.

**Fig 1:**
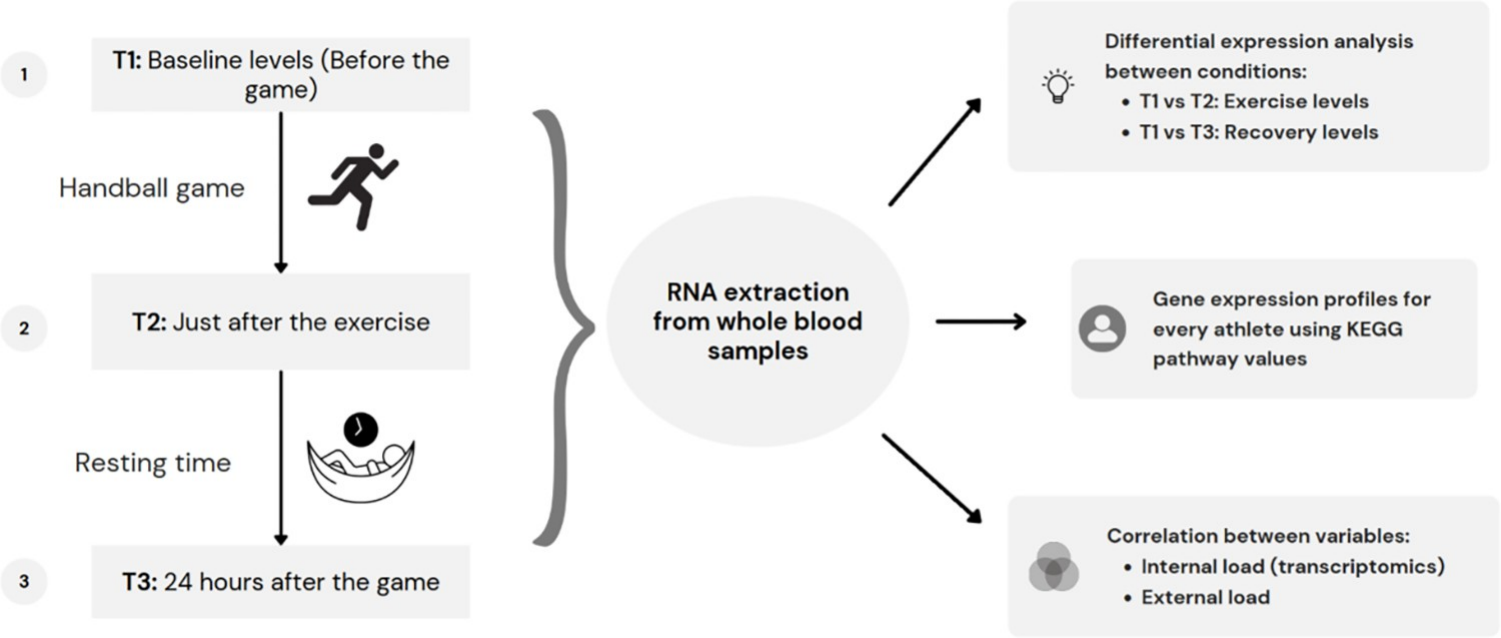
Flowchart of the experimental design followed in this study.

The experimental design was built under a paired design analysis due to the nature of the obtained samples, which comprised paired data. Each observation in one condition had a corresponding match in the other condition. As all individuals contributed samples across all conditions, this design was employed to control variability between individuals (Fig 1).

Thus, the baseline level samples at T1 were compared against the gene expression values extracted from the samples obtained after the match at T2 to determine which genes were the most differentially expressed between the two conditions (T2 –T1). The baseline levels were then compared with samples obtained 24 h after finishing the match at T3, inferring the genes with different expression values and tracking the recovery levels of the athletes. (T3 –T1).

Subsequently, a KEGG pathway score was computed using the gene values from each metabolic pathway in all individuals for each condition. The scores of different KEGG pathways were correlated with variables calculating the external load, encompassing both acute and chronic response variables.

### Session monitoring

This study was conducted using the WIMU PRO™ system (RealTrack Systems SL, Almería, Spain). Each device, whose dimensions were 81x45x16 mm (height/width/depth) and weighed 70 g, was fitted to the back of each player with an adjustable vest (Rasán®, Valencia, Spain).

The match consisted of two 30-minute periods with a10-minutes rest. In contrast to other sports, one of the characteristics of handball is that players can be substituted interchangeably without stopping all players from playing the same number of minutes. The offensive and defensive systems of play vary. Still, the team played the match with a 60 defense (six players close to the defensive area) and offensively with a 33 system (two rows of three players).

The positioning data record was monitored in real-time and subsequently analyzed using SPRO^TM^ software version 946–949 (SPRO™, RealTrack Systems, 2018). The system operates using triangulations between four antennas with patented ultra-wideband technology (18 Hz sampling frequency) placed 5 m away from each corner of the court and at a height of 6 m. These units included several sensors recorded at different sampling frequencies. The sampling frequencies used for the 3-axis accelerometer, gyroscope, and magnetometer were 100 Hz and 120 kPa for the barometer [25].

High-speed running (HSR, distance covered in meters above 18.1 kph) and the total number of high-intensity accelerations (HIA) (acc +2), high-intensity deceleration (HID) (dec +2) (in m·s-2), high-intensity acceleration per min (acc +2/min), and high-intensity deceleration per min (dec +2/min) (m·s-2·min-1) were recorded [26]. HIA and HID were defined as events above 2 g and were extracted from the root data reported by the system using the SPRO^TM^ software [27].

### RNA collection and sequencing

2.5 ml of blood was drawn from each athlete, collected in a PAXgene Blood RNA tube (QIAGEN GmbH, Germany), and stored at -80°C until RNA extraction was performed according to the manufacturer’s instructions. All blood samples were collected at FC Barcelona facilities. Subsequently, RNA was sequenced using Illumina technology with the Illumina TruSeq Sample Prep Kit and sequenced on a NovaSeq 6000 Sequencing System (Illumina).

The sequenced 75-base pair-long paired-end reads were mapped to the GRCh38 reference genome. Once these reads were mapped, the genes defined in ENSEMBL annotation were used. Thus, 14,235 genes were available for further analysis. A standard quality control measure was applied to the RNA, verifying both RNA integrity and the quality of the library kit preparation.

### Normalization and filtering of poorly expressed genes

**Fig 2:**
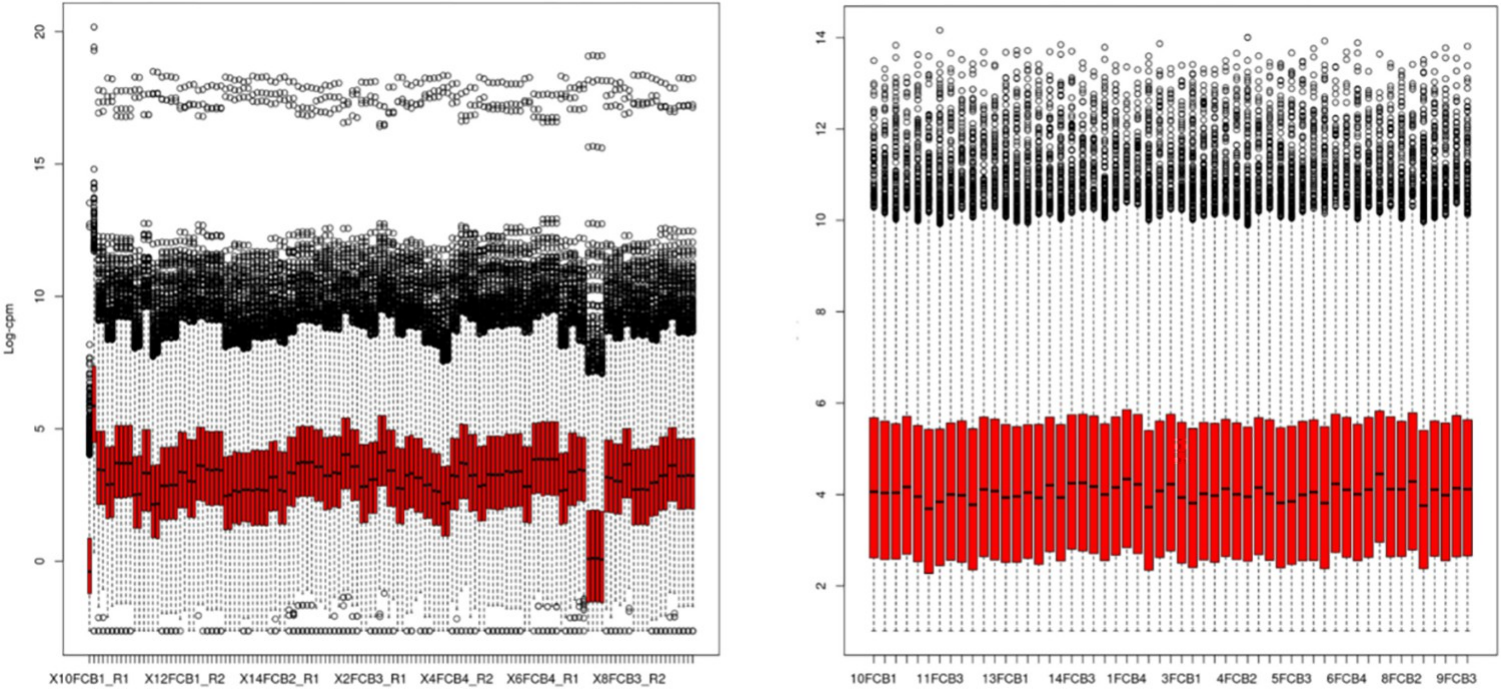
Normalization Comparison—On the left, unnormalized counts; on the right, counts normalized by library size.

Normalization was conducted for all samples using scaling factors to convert the raw library sizes to effective library sizes. Each sample was corrected using this normalization factor and the results were included in the analysis (Fig 2). In addition, poorly expressed genes were filtered to retain only those genes that had a good minimum number of reads.

### Ethical considerations

The study was carried out by Beyond You, Institut de Recerca de l’Hospital Sant Pau and Futbol Club Barcelona (FCB, Barcelona, Spain) following relevant guidelines and regulations. Institutional board approval for the study was obtained from the Ethics Commission of the Consell Català de l’Esport (Code 012/CEICEGC/2021, Generalitat de Catalunya, Barcelona, Spain). Written informed consent was collected and all data were anonymized to ensure confidentiality. All procedures involving human participants followed the ethical standards of the institutional and/or national research committee and with the 1964 Helsinki Declaration and its later amendments or comparable ethical standards. This study involved the collection of daily EPTS data and blood samples at three-time points from professional handball players of a male team (see Fig 1). The samples were obtained across two consecutive days, capturing data at three distinct time points. Specifically, on the first day (1^st^ of June, 2021), samples were collected at 13:30 (T1) before the match, followed by an additional collection at 17:30 (T2) after the match. The process continued on the subsequent day (2^nd^ of June, 2021) with a single sample collection at 13:30 (T3), 24 hours after the match.

### Statistical analysis

Differential expression analysis, GO, and KEGG pathway enrichment, as well as linear correlations between gene expression levels and external load values, were conducted. To mitigate false positives and address multiple comparison effects for all analyses, we employed the False Discovery Rate (FDR) technique [28]. All p-values have also been adjusted using the False Discovery Rate (FDR) method. The pipelines for these analyses were developed using the R programming language.

### Differential expression analysis

Gene expression has been quantified using salmon [29]. Salmon uses algorithms to provide accurate RNA expression estimates values and performs its inference using an expressive model of RNA-seq data that takes into account experimental attributes and biases commonly observed in *real* RNA-seq data. Then, a differential expression analysis was performed between conditions, and a linear regression model was built using limma package [30]. Limma is a software package designed for gene expression data analysis from microarray or RNA-seq technologies that employs linear models for evaluating differential expression in complex experimental setups with multiple factors. The model utilized for analysis is defined as follows:

Model = 0 + Time + Ind

Here, ’0’ signifies the absence of an intercept, ’Time’ denotes the sample condition (baseline, immediately post-race, or 24 hours after), while ’Ind’ accounts for inter-individual variability encompassing variables such as height, weight, age, and athlete position.

Subsequent to obtaining the list of Differentially Expressed Genes (DEGs) from the aforementioned model, enrichment analyses encompassing Gene Ontology (GO) and Kyoto Encyclopedia of Genes and Genomes (KEGG) were conducted. To perform these analyses, the GOstats package was employed [31]. This facilitated the identification of body systems enriched in the two comparisons, assessing both over- and under-representation of respective GO terms. Additionally, a conditional hypergeometric test was utilized to unveil relationships among the GO terms.

Moreover, GO terms based on gene size and enrichment by DE genes were filtered to prioritize more reliable and interesting terms. Terms involving few genes may be less reliable, while large terms could offer limited insights. A minimum value on the Count and Size columns and a maximum count value was used for this filtering process. The GOplot package was used to visualize the results [32].

Finally, the DEG list was used to identify the enriched KEGG pathways. In this case, the hypergeometric test was conducted using the signatureSearch package [33].

### Correlation between the internal and external loads

The complete gene set extracted from whole blood samples in each condition was used to compute an abnormality score for the 323 pathways stored in the KEGG pathway database using the CPM values obtained from the salmon pipeline.

The external and internal loads were correlated using the ggstatsplot package [34]. These correlations were executed using Pearson correlation analysis. Pearson’s correlation test explores relationships between two continuous variables. This method is frequently utilized for numerical variables and assigns a value ranging between −1 and 1. In this scale, 0 indicates no correlation, 1 represents a complete positive correlation, and −1 indicates a total negative correlation. The p-value was adjusted for multiple comparisons using the FDR algorithm. Therefore, in this case, the scores of the 323 pathways were correlated with the variables representing external load obtained using EPTS, as described in the Materials and Methods section.

## Results

### Differential expression analysis

**Fig 3:**
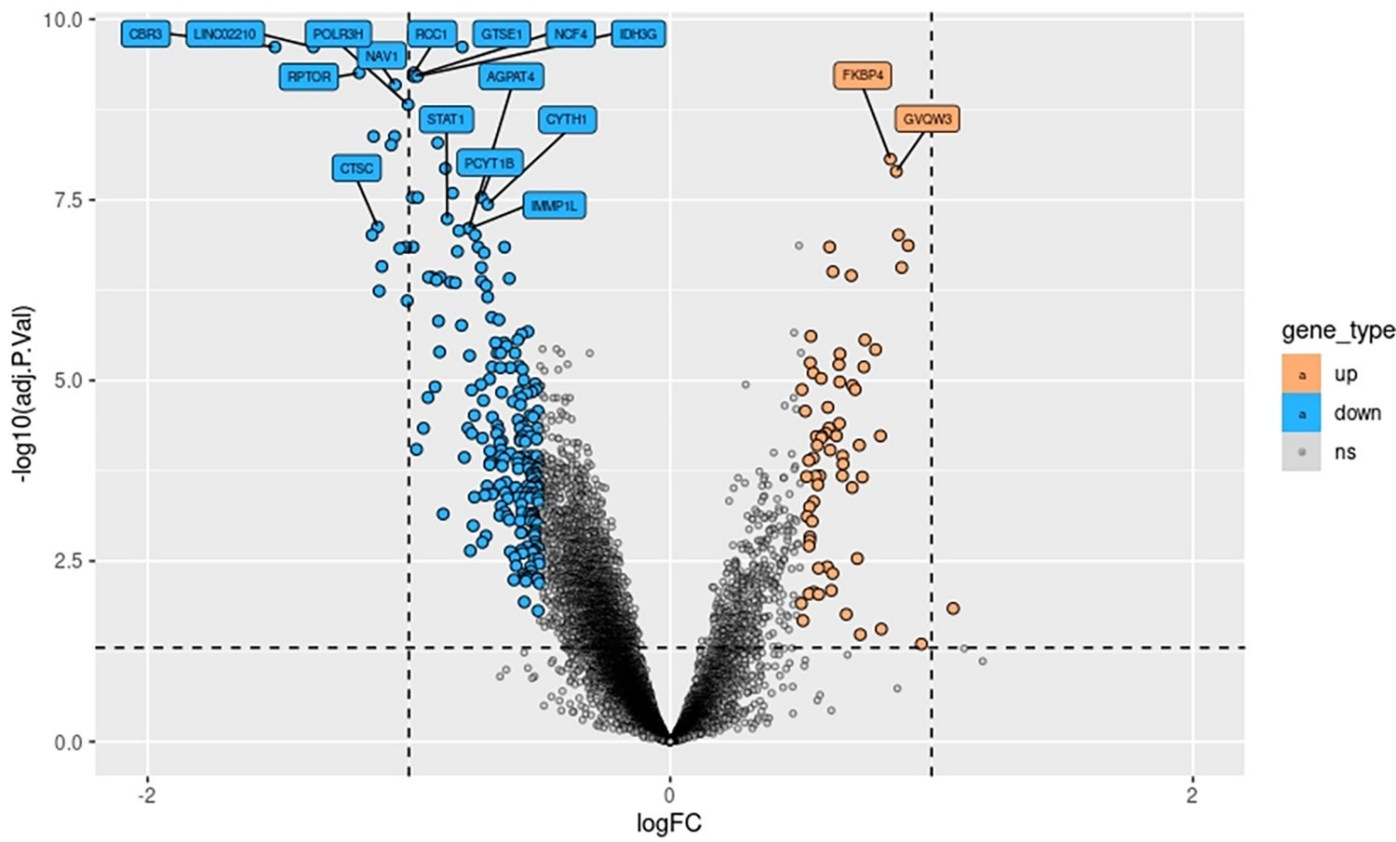
Volcano plot showing the differential expressed genes in Comparison 1. The top 20 genes (higher adjusted p.value) are the ones labeled. Log fold change LogFC is represented on X axis and–log10 adj.p.value on the Y axis. A log(FC) of 1 means twice as expressed.

A total of 3,717 of the 14,632 total genes detected were found to be differentially expressed compared to baseline levels just after finishing the match (Comparison 1) (Fig 3). Of these genes, 3,234 were downregulated after playing the match and 483 were upregulated. However, not significant differences in gene expression were found between baseline levels (T1) and levels 24 h after the exercise (T2).

**Fig 4:**
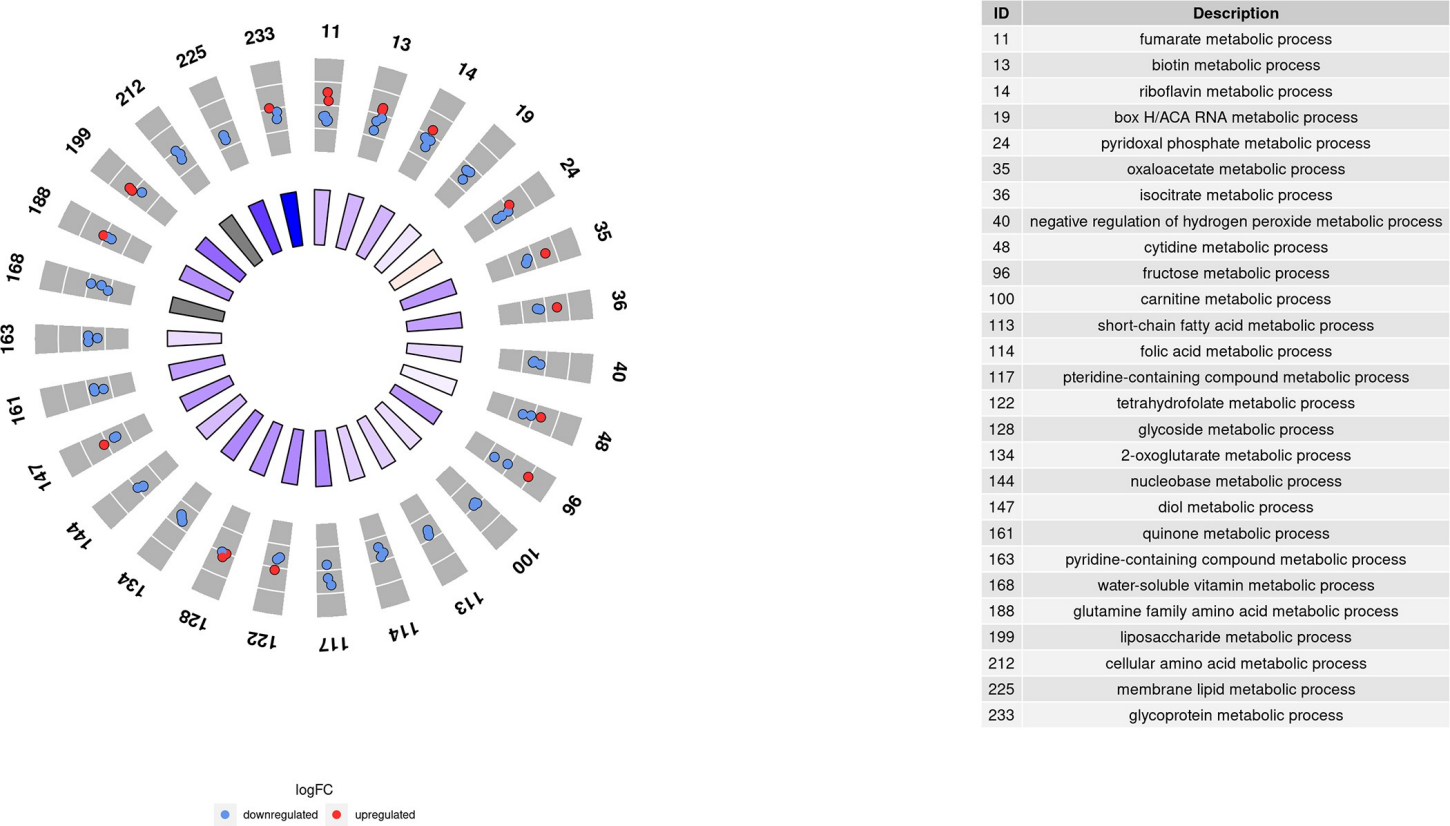
Enriched GO terms related to metabolic processes in comparison 1.

**Fig 5:**
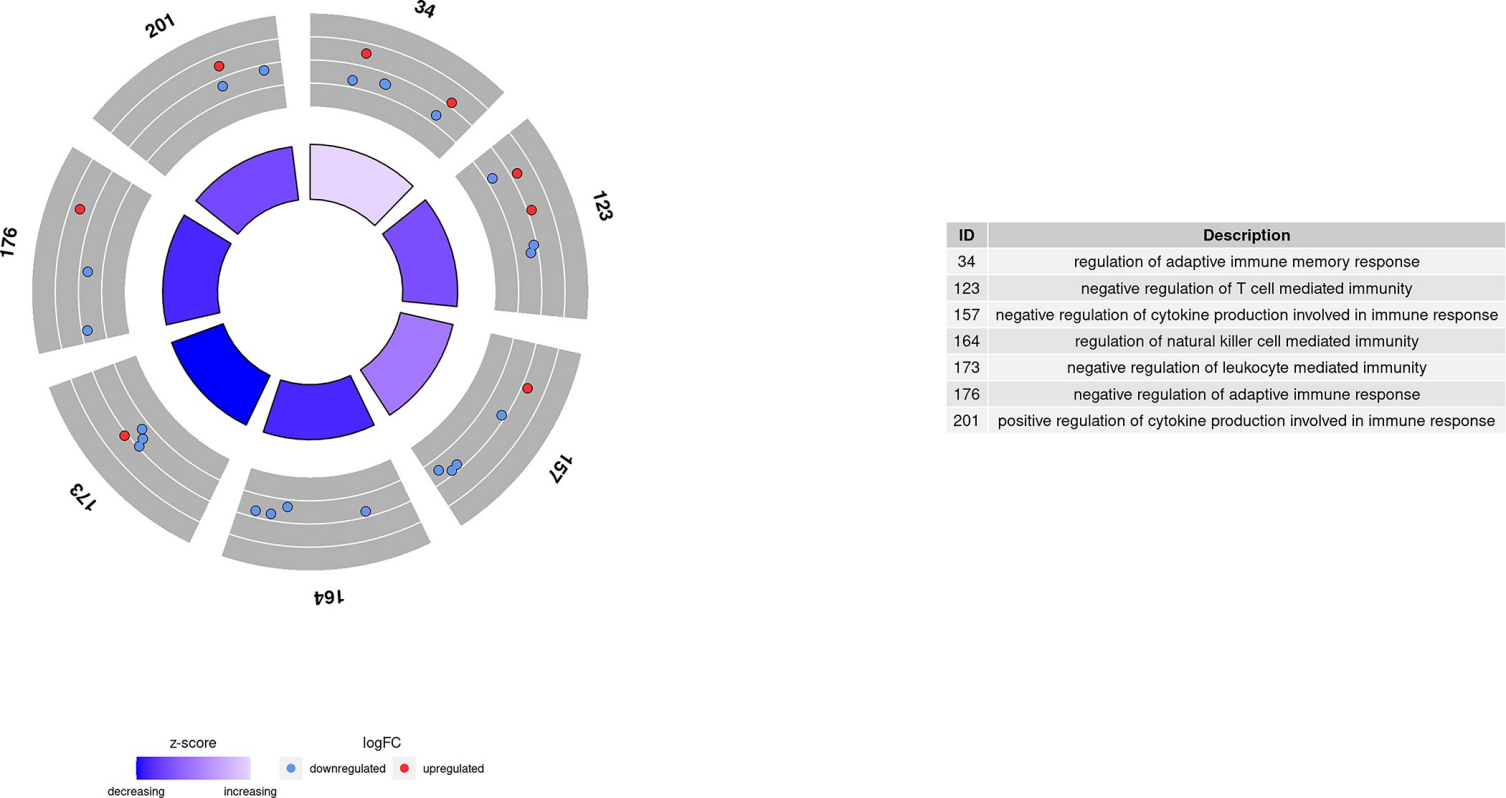
Enriched GO terms related to immune processes in comparison 1.

**Fig 6:**
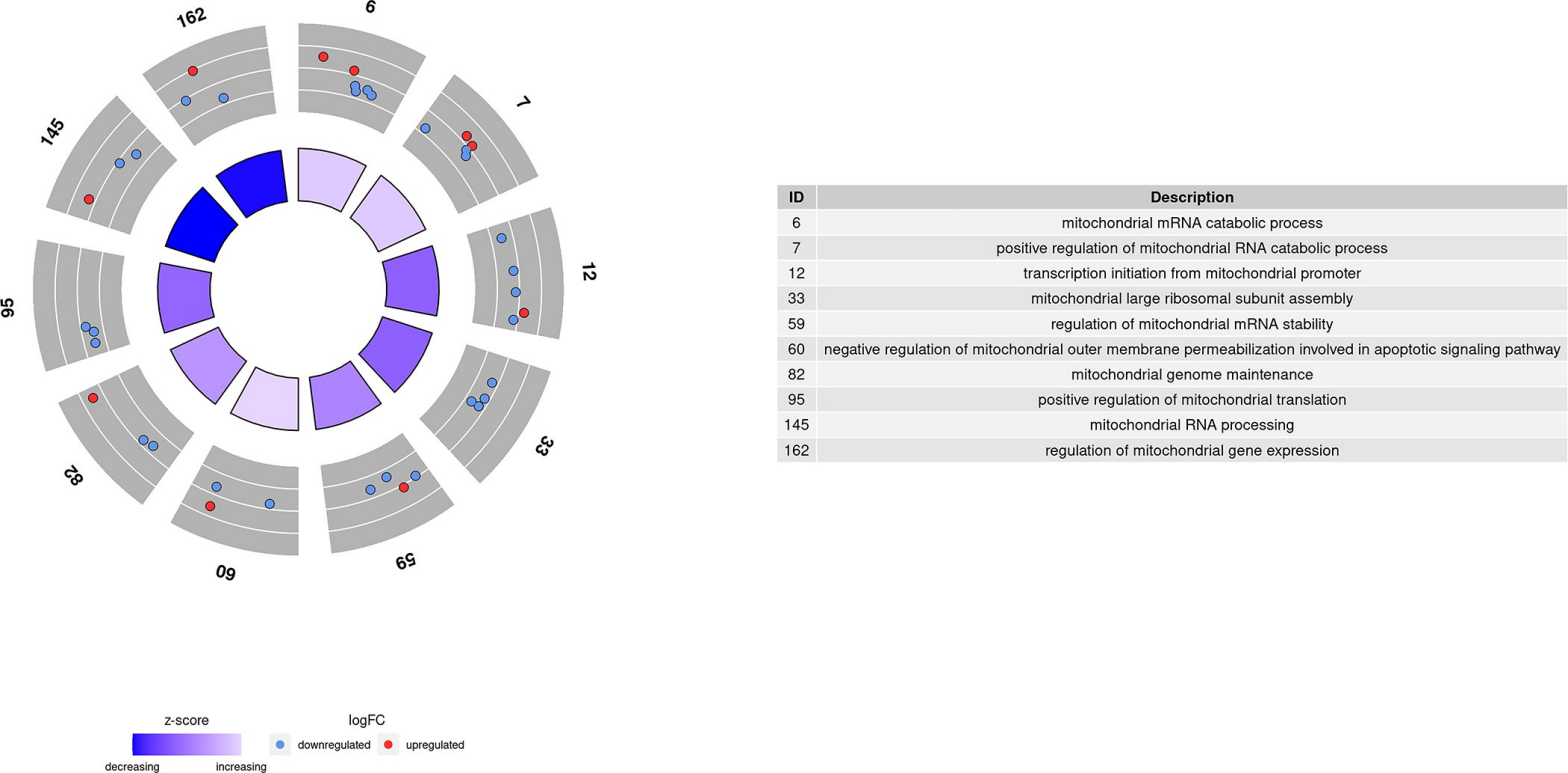
Enriched GO terms related to mitochondrial processes in comparison 1.

Those 3,717 DEGs obtained in comparison 1 were used as inputs for GO term and KEGG pathway enrichment analysis. As a result, 233 GO terms were significantly enriched with a p-value adjusted to < 0.05 after performing a conditional analysis, filtered by size and weight. These GO terms can be grouped into three main systems: mitochondrial system, metabolic processes, and immune system. As shown in Figs 4–6, many of the genes were downregulated after exercise, and therefore, most of the GO terms had a decreasing global Z-score. Only some mitochondrial GO terms and metabolic processes had an increasing Z-score.

The same enrichment procedure was performed for KEGG pathways using the DEG list obtained from Comparison 1. This analysis revealed 18 KEGG pathways that were associated with various biological processes. Among these pathways were folate metabolism, biosynthesis of cofactors, glycosaminoglycan metabolism, and the metabolism of branched-chain amino acids (valine, leucine, and isoleucine).

Furthermore, pathways linked to cell regulation (mismatch repair, nucleotide excision repair, DNA replication, and apoptosis) were identified (Table 2). Another interesting cluster of pathways included the citrate cycle (TCA) and 2-Oxocarboxylic acid metabolism pathways, which are responsible for energy generation.

**Table 2:** List of the KEGG pathways enriched in comparison 1.

KEGG ID	Description	p-value	p-value adjusted
**hsa04141**	Protein processing in endoplasmic reticulum	0.000000289 0.000000403	0.00006513
**hsa01240**	Biosynthesis of cofactors	4.03e-07	0.00006513
**hsa03030**	DNA replication	0.00000611	0.00006579
**hsa03013**	Nucleocytoplasmic transport	0.00000117	0.00009457
**hsa00020**	Citrate cycle (TCA cycle)	0.0000167	0.00108
**hsa03430**	Mismatch repair	0.00025	0.01383
**hsa00280**	Valine, leucine and isoleucine degradation	0.00046	0.02166
**hsa04210**	Apoptosis	0.00053	0.02166
**hsa04931**	Insulin resistance	0.00066	0.02384
**hsa01210**	2-Oxocarboxylic acid metabolism	0.00077	0.02518
**hsa03420**	Nucleotide excision repair	0.00104	0.02808
**hsa04146**	Peroxisome	0.00108	0.02808
**hsa03060**	Protein export	0.00119	0.02808
**hsa00532**	Glycosaminoglycan biosynthesis–chondroitin sulfate / dermatan sulfate	0.0013	0.02808
**hsa00670**	One carbon pool by folate	0.0013	0.02808
**hsa04142**	Lysosome	0.00215	0.04027
**hsa04140**	Autophagy—animal	0.00224	0.04027
**hsa03018**	RNA degradation	0.003	0.04881

### Correlation between the internal and external loads

After compiling all data from the external load, it was organized into two distinct tables, as shown in S1 and S2 Tables. The first table comprises the average values of the external load variables throughout the season, indicating the chronic response, whereas the second table displays the results of the variables obtained during matches, representing the acute response. Then, gene expression values will be correlated with the external load data (S1 and S2 Tables) in the downstream analyses.

#### Acute response correlation

First, we aimed to elucidate the acute physiological response of the body by correlating the metabolic pathway scores immediately and 24 h after the conclusion of the match with the corresponding external load imposed on each player.

**Fig 7:**
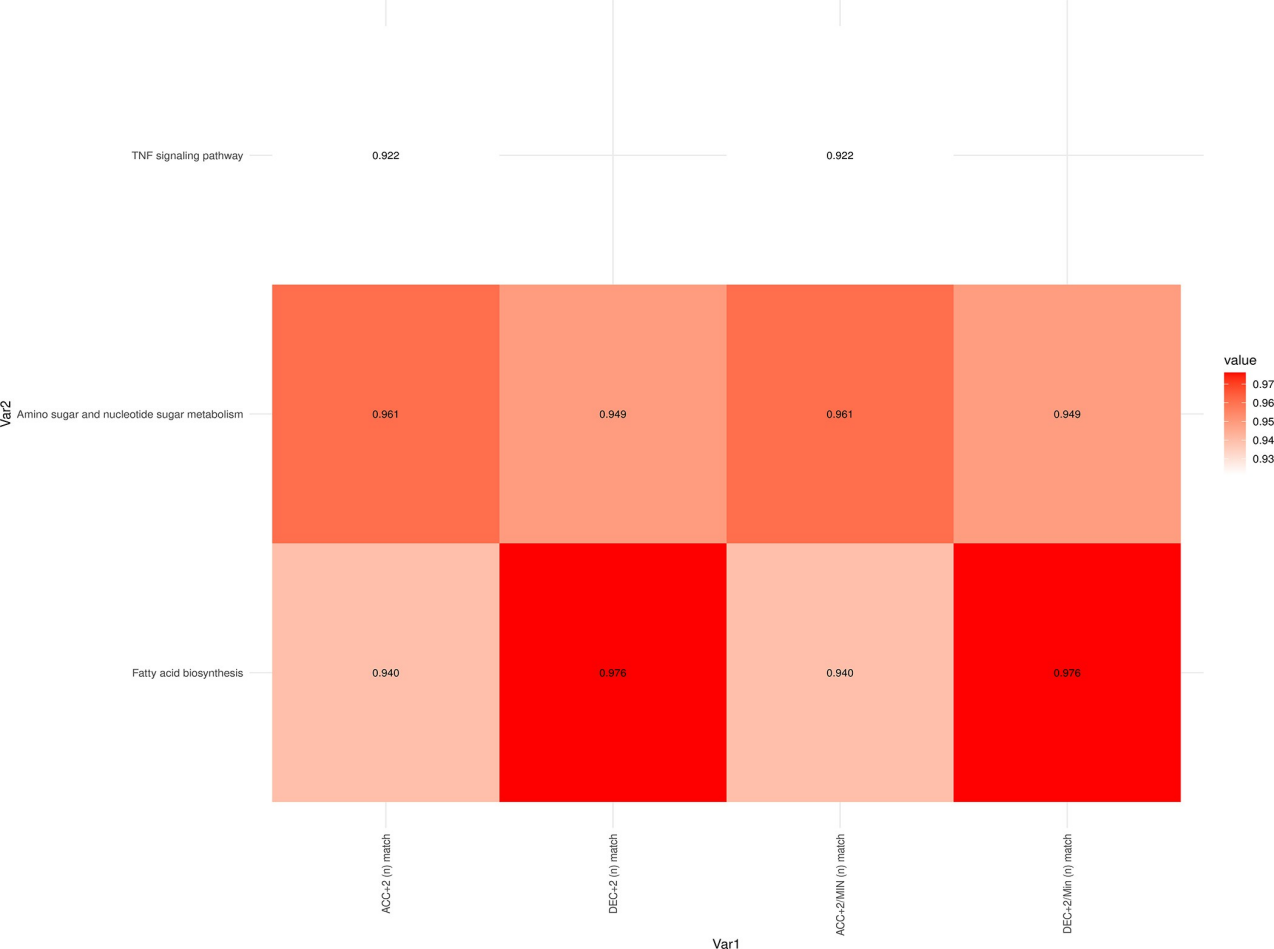
Correlation values between internal and external load match variables at baseline levels (Time 1). All correlation values shown are statistically significant, with an adjusted p-value < 0.05.

Before the match at baseline levels (T1), correlations were observed between fatty acid biosynthesis, amino sugar and nucleotide sugar metabolism, and the TFN signaling pathway with both acceleration (ACC +2) and deceleration (DEC +2) values. (Fig 7, S3 Table)

**Fig 8:**
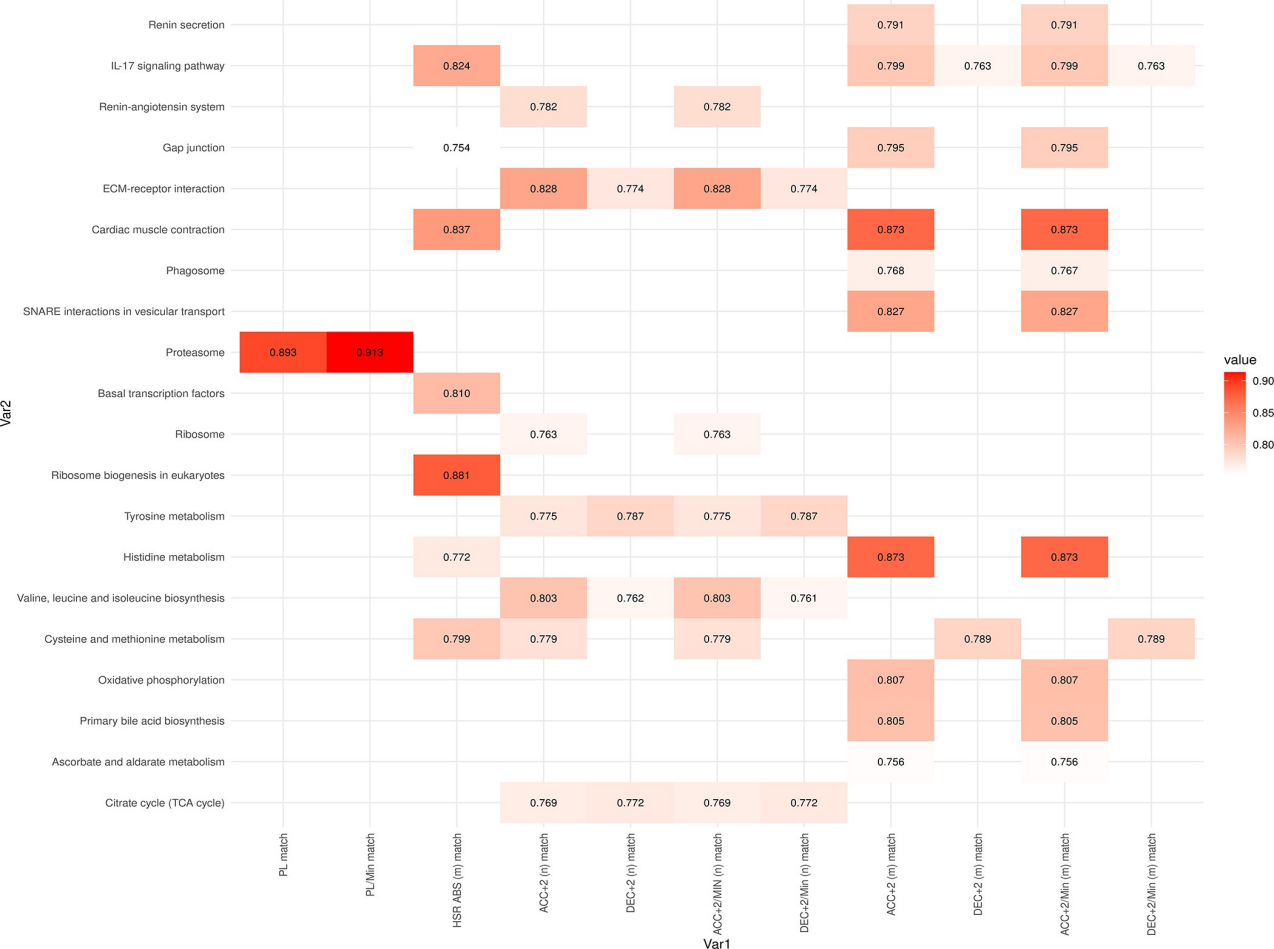
Correlation values between internal and external load variables after match (Time 2). All correlation values shown are statistically significant, with an adjusted p-value < 0.05.

At T2, just after the match, (Fig 8, S4 Table) external load showed significant correlations with several metabolic pathways, including proteasome pathway levels, valine, leucine, and isoleucine biosynthesis, tyrosine metabolism, cysteine and methionine metabolism, and histidine metabolism. Correlations were also observed with pathways related to the oxidative environment, such as ascorbate and aldarate metabolism, oxidative phosphorylation, and energy-related pathways, like the citrate cycle. Moreover, cellular processes’ regulation, such as basal transcription factors, phagosomes, ECM-receptor interactions, and gap junctions, correlated with ACC and DEC values.

**Fig 9:**
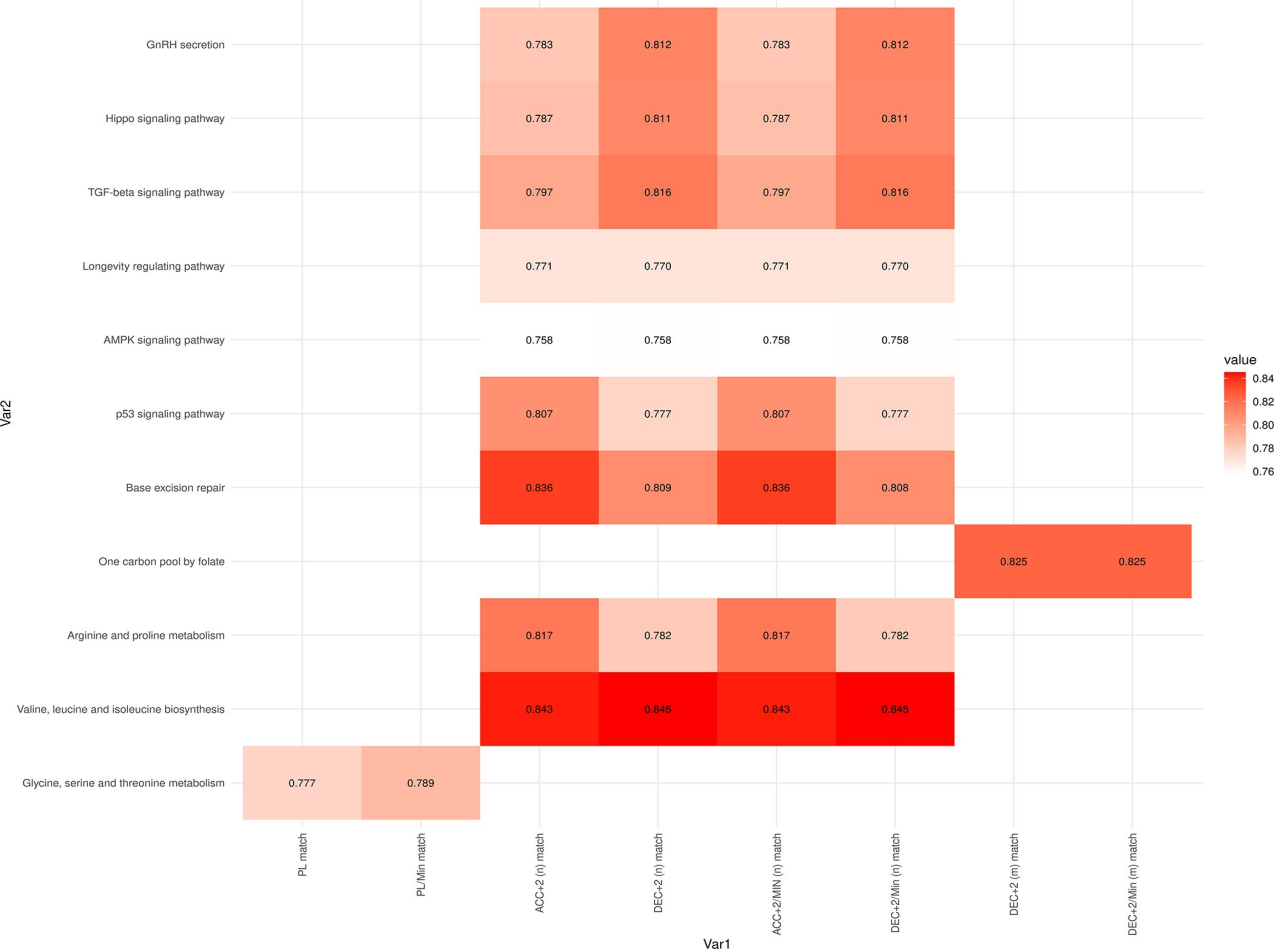
Correlation values between internal and external load match variables 24 hours after finishing the match (Time 3). All correlation values shown are statistically significant, with an adjusted p-value < 0.05.

At T3, glycine, serine, and threonine metabolism correlated with PL and PL/min values, while other amino acids such as valine, leucine, isoleucine, arginine, and proline metabolism pathways were linked with ACC and DEC values (Fig 9, S5 Table). Additionally, correlations were observed with pathways related to repair mechanisms, inflammation (TFG-beta signaling pathway), regulation (AMPK signaling pathway and p53 signaling pathway) and GNRH secretion, all of which correlated with ACC and DEC values.

#### Chronic response correlation

Next, we aimed to describe the body’s chronic response by correlating metabolic pathway scores immediately and 24 hours after the match conclusion with the corresponding season average external load for each player.

**Fig 10:**
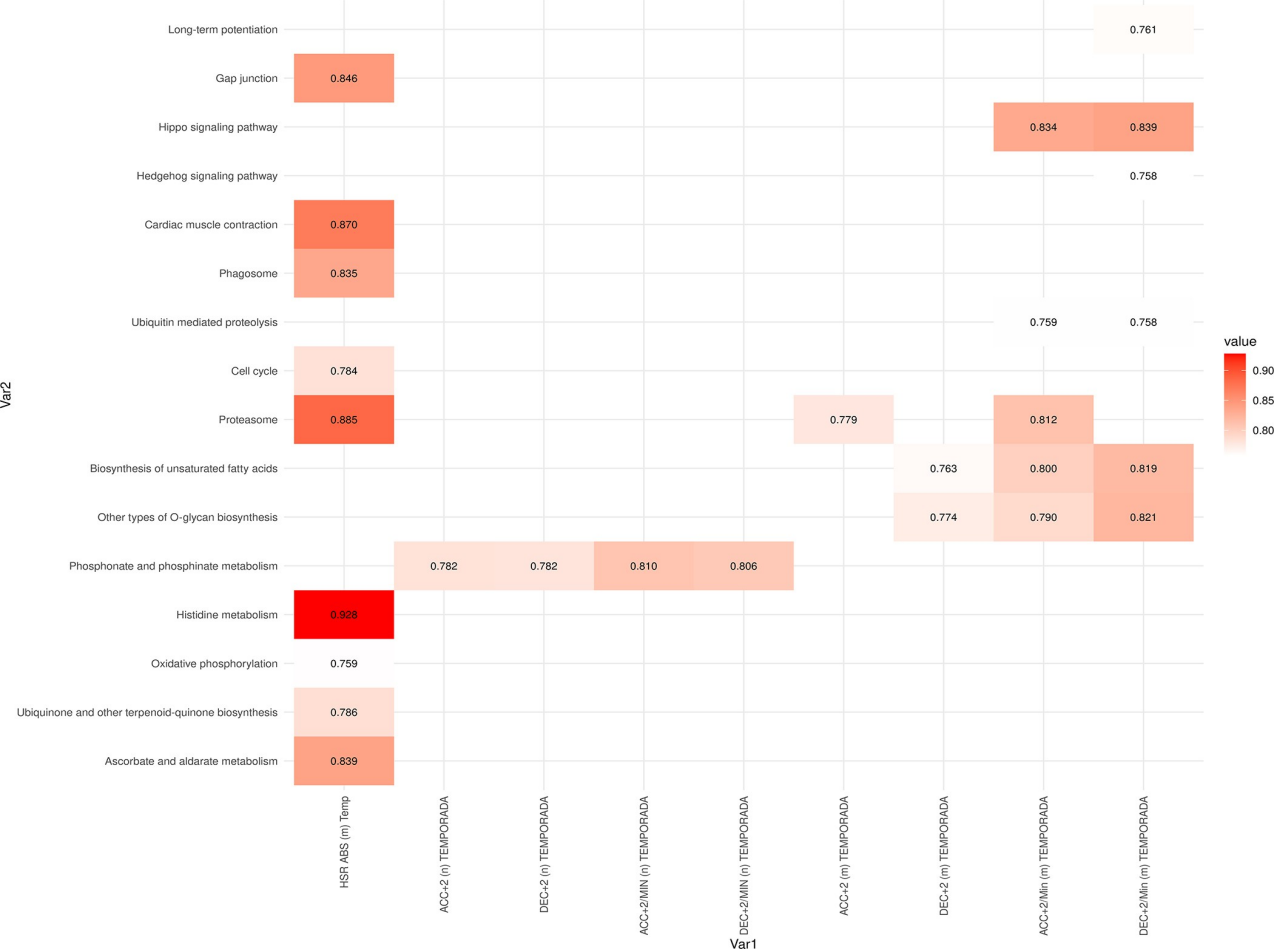
Correlation values between internal and external load season average variables just after finishing the match (Time 2). All correlation values shown are statistically significant, with an adjusted p-value < 0.05.

At T1, there were no correlations between seasonal average external load variables and baseline pathway levels. At T2, HSR absolute season average values were correlated with pathways related to amino acid metabolism, such as histidine metabolism (Fig 10, S6 Table). Other significant correlations were found with pathways like ascorbate and aldarate metabolism, cardiac muscle contraction, and proteasomes and phagosomes. Moreover, ACC and DEC variables were significantly correlated with phosphonate and phosphine metabolism, as well as the biosynthesis of unsaturated fatty acids, among others.

**Fig 11:**
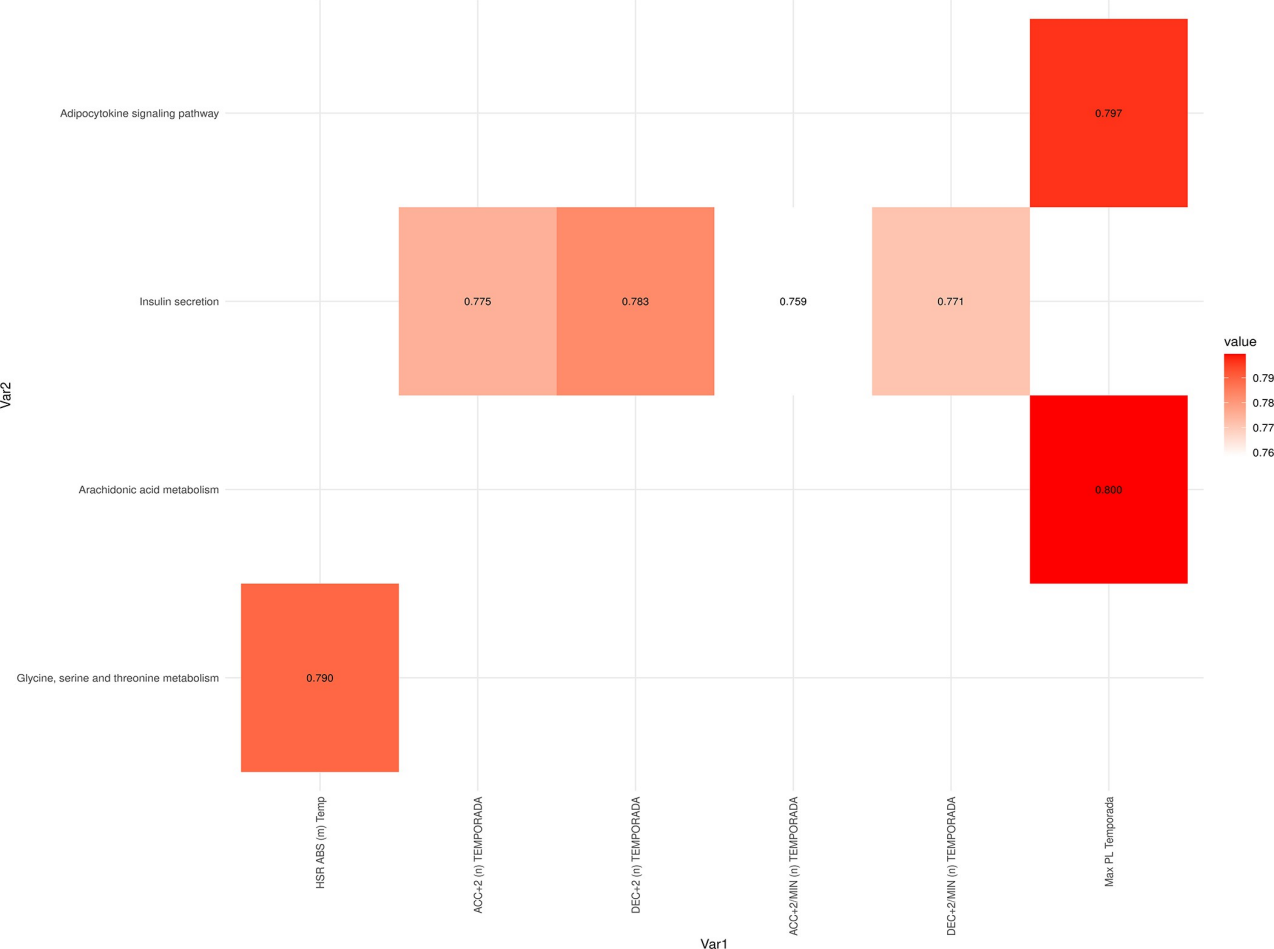
Correlation values between internal and external load season average variables 24 hours after finishing the match (Time 3). All correlation values shown are statistically significant, with an adjusted p-value < 0.05.

Finally, at T3, the absolute HSR value was significantly correlated with glycine, serine, and threonine metabolism (Fig 11, S7 Table). The ACC and DEC values were correlated with insulin secretion, and the maximum PL of the season was correlated with the adipocytokine signaling pathway and arachidonic acid metabolism.

## Discussion

This study aims to comprehend the impact of handball practice on sub-elite athletes by investigating transcriptomic changes occurring during a match. It involves identifying and characterizing these transcriptomic changes and establishing correlations between gene expression and external load measured through Electronic Performance and Tracking Systems (EPTS variables). Ultimately, this comprehensive analysis seeks to evaluate both acute and chronic responses to exercise within the context of a handball match.

The findings provide compelling evidence that 25% of the total analyzed genes exhibited significant differences in expression in Comparison 1. The majority of genes were downregulated, suggesting that their levels were significantly lower after the match compared to the baseline condition. Furthermore, there was an enrichment observed in Gene Ontology (GO) terms and KEGG pathways related to mitochondrial, immune, and metabolic systems during the exercise performed by elite athletes. Additionally, significant correlations have been observed between gene expression levels (internal load) and external load variables assessing both acute and chronic responses during a handball match.

### Conjoint analysis of internal and external loads

#### Acute response

At T2, numerous pathways linked to metabolism and biosynthesis of amino acids, specifically to branched-chain amino acids, tyrosine, histidine, cysteine, and methionine exhibited a significant correlation with ACC and DEC variables. These amino acids are commonly utilized as primary supplements for athletes, emphasizing the significance of these findings.

Previous studies have demonstrated that oral tyrosine supplementation enhances exercise capacity in hot environments [35]. Additionally, supplementation with branched-chain amino acids (BCAAs) has shown to protect against muscle damage by lowering creatine kinase activity and potentially mitigating muscle damage within the initial 24 hours post-exercise [36]. This supports the notion that consistent daily doses of BCAAs are more beneficial for athlete recovery compared to intermittent doses [37]. Lastly, cysteine supplementation has exhibited positive effects in reducing exercise-induced fatigue, possibly attributed to increased fatty acid utilization [38].

The relationship between these different amino acids importance in exercise supplementation with variables measuring intensity of the athletes during matches provides valuable information about athletes’ performance during exercise enhancing future performance through supplementation.

At T3, several pathways related to oxidative stress (TFG-beta signaling pathway, AMPK signaling pathway and one-carbon pool by folate pathway) correlated with variables linked to high-intensity actions: ACC and DEC.

These two pathways have previously been associated with physical activity. The TFG-beta signaling pathway, previously known for elevating TGF-β activity in skeletal muscle, inhibits the enhancement of mitochondrial fuel oxidation after training and restrains the increase in insulin sensitivity [39]. This relationship is connected to the AMPK signaling pathway, responsible for regulating metabolism and preserving mitochondrial homeostasis [40]. In addition, the one-carbon pool by folate pathway is important for mitigating oxidative stress and amino acid homeostasis, specifically the metabolism of glycine, serine, and methionine [41].

Finally, glycine, serine, and threonine metabolism pathways levels correlated with player’s external load (PL) and PL/min from the match, supporting the idea that these amino acids could directly impact on the recovery of athletes after aerobic exercise.

### 2) Chronic response

The average season values of HSR were found to correlate with several pathways including Histidine metabolism, ascorbate and aldarate metabolism (vitamin C), cardiac muscle contraction, proteasome and phagosome, ubiquinone, and oxidative phosphorylation pathways at T2. This is particularly relevant since previous studies have linked HSR to the risk of injury [42]. Therefore, the various pathways correlated with HSR values are associated with exercise performance.

Histidine and histidine-related compounds (HRCs) have been indicated to enhance anaerobic exercise performance by stabilizing pH and inhibiting glycolysis [43]. Additionally, vitamin C metabolism plays a role in maintaining bone, muscle, and tendon integrity, reducing oxidative stress (crucial post-exercise), and is connected to collagen, a primary component of tendons [44]. So, further studies of these variables could provide important information regarding injury risk.

Finally, at T3, ACC and DEC showed significant correlation with insulin secretion meanwhile the maximum PL of the season correlated significantly with the adipocytokine signaling pathway. The well-known relationship between insulin and exercise is understood, as insulin’s fuel storage effects must be suppressed during physical activity [45,46]. Moreover, adipocytokines play a central role in regulating insulin resistance, inflammation, and immunity [47] leading to the conclusion that insulin secretion is associated with external load at a chronic response level.

#### Practical application

From a practical applications point of view, these correlations can provide insights for future personalized treatments and individual recommendations for athletes’ preparation, training, and recovery. Utilizing transcriptomic information enables the evaluation of oxidative stress, inflammatory responses, and mitochondrial pathways for each athlete.

For instance, the significant discovery linking branched-chain amino acids with variables measuring external load like ACC, DEC, and HSR can tailor the intake of supplementation for athletes. Another crucial practical application involves the correlation between several metabolic pathways and external load variables, aiding sports nutritionists in developing personalized nutrition plans for each athlete. Additionally, the information acquired from measuring oxidative environment pathways and the subsequent ability to correlate it with external load variables can assist in detecting the oxidative levels of each athlete and being a critical information for the utilization of antioxidant compounds through supplementation during the recovery phase to protect the body from oxidative stress. Therefore, these findings enable the customization of an athlete’s recovery phase after exercise and can assist the team’s physical trainers and medical staff in making decisions concerning the athlete.

These discoveries pave the way for a comprehensive personalized monitoring of athletes before, during, and after a match, considering both external and internal factors along with their correlations.

## Conclusions

This investigation marks a significant breakthrough in sports science by unraveling the intricate relationship between external load and transcriptomic internal load in professional handball players, and the identification of transcriptomic alterations during handball competition. The study linked pathways related to amino acids, inflammation, oxidative stress, and regulation with external load variables like HSR, ACC and DEC during a handball match and its subsequent recovery. Notably, the pathways related to the biosynthesis of branched-chain amino acid pathways, including valine, leucine, and isoleucine as well as histidine metabolism, demonstrate particular significance. Moreover, the research reveals dysregulation in the immune system, mitochondrial functions, and various metabolic pathways during the match.

This information will serve to establish criteria for improving and generate personalized nutritional and training guidelines for elite athletes based on transcriptomic data, thereby enhancing their performance and facilitating optimal recovery.

## Supporting information

S1 Table External load variables collected during the match. (DOCX)

S2 Table Average of the External load variables collected during the whole season. (DOCX)

S3 Table Correlation values between internal and external load match variables at baseline levels (Time 1). (DOCX)

S4 Table Correlation values between internal and external load variables after match (Time 2). (DOCX)

S5 Table Correlation values between internal and external load match variables 24 hours after finishing the match (Time 3). (DOCX)

S6 Table Correlation values between internal and external load season average variables just after finishing the match (Time 2). (DOCX)

S7 Table Correlation values between internal and external load season average variables 24 hours after finishing the match (Time 3). (DOCX)
