# Supplementary material for: Unraveling athletic performance: Transcriptomics and external load monitoring in handball competition
Source: PLoS One. 2024 Mar 11;19(3):e0299556. doi: 10.1371/journal.pone.0299556 (PMC10927131; doi:10.1371/journal.pone.0299556)
Supplement: S1 Table — (DOCX) [file pone.0299556.s001.docx]

**Table S1**: External load variables collected during the match

|  | **1FCB** | **5FCB** | **6FCB** | **7FCB** | **8FCB** | **9FCB** | **10FCB** | **11FCB** | **12FCB** | **14FCB** |
| --- | --- | --- | --- | --- | --- | --- | --- | --- | --- | --- |
| **PL match** | 67.2 | 77.39 | 93.52 | 84.53 | 75.35 | 58.95 | 57.15 | 64.79 | 59.35 | 46.45 |
| **PL/Min match** | 1.09 | 1.26 | 1.58 | 1.38 | 1.23 | 0.93 | 0.93 | 1.05 | 0.97 | 0.75 |
| **HSR ABS (m) match** | 43.89 | 530.8 | 729.4 | 218.31 | 158.58 | 169.83 | 29.9 | 60.42 | 342.78 | 528.16 |
| **HSR Rel (m) match** | 0 | 92.18 | 27.1 | 0 | 0 | 0 | 0 | 0 | 0 | 56.38 |
| **Match Distance** | 5127.6 | 5578.2 | 6290.9 | 5363.9 | 5376.3 | 5078.3 | 4481.7 | 5227 | 5334.9 | 2031.2 |
| **Distance/Min match** | 83.80 | 91.17 | 102.82 | 87.66 | 87.85 | 83.00 | 73.23 | 85.40 | 87.19 | 33.18 |
| **ACC+2 (n) match** | 72 | 121 | 138 | 130 | 131 | 200 | 60 | 123 | 135 | 342 |
| **DEC+2 (n) match** | 62 | 94 | 82 | 108 | 104 | 186 | 50 | 85 | 88 | 307 |
| **ACC+2/MIN (n) match** | 1.17 | 1.97 | 2.25 | 2.12 | 2.14 | 3.26 | 0.98 | 2.01 | 2.20 | 5.58 |
| **DEC+2/Min (n) match** | 1.01 | 1.53 | 1.34 | 1.76 | 1.7 | 3.04 | 0.81 | 1.38 | 1.43 | 5.01 |
| **ACC+2 (m) match** | 333.11 | 848.42 | 1119.7 | 541.22 | 685.2 | 601.15 | 249.24 | 523.13 | 698.51 | 546.23 |
| **DEC+2 (m) match** | 279.94 | 611.97 | 656.94 | 435.83 | 483.37 | 560.62 | 229.46 | 299.46 | 360.73 | 501.93 |
| **ACC+2/Min (m) match** | 5.44 | 13.86 | 18.30 | 8.84 | 11.2 | 9.82 | 4.07 | 8.54 | 11.41 | 8.92 |
| **DEC+2/Min (m) match** | 4.57 | 10.00 | 10.73 | 7.12 | 7.9 | 9.16 | 3.74 | 4.89 | 5.89 | 8.20 |
