# Supplementary material for: Unraveling athletic performance: Transcriptomics and external load monitoring in handball competition
Source: PLoS One. 2024 Mar 11;19(3):e0299556. doi: 10.1371/journal.pone.0299556 (PMC10927131; doi:10.1371/journal.pone.0299556)
Supplement: S2 Table — (DOCX) [file pone.0299556.s002.docx]

**Table S2:** Average of the External load variables collected during the whole season

|  | **1FCB** | **5FCB** | **6FCB** | **7FCB** | **8FCB** | **9FCB** | **10FCB** | **11FCB** | **12FCB** | **14FCB** |
| --- | --- | --- | --- | --- | --- | --- | --- | --- | --- | --- |
| **Average RPE season** | 6.29 | 6.14 | 7.57 | 8.43 | 8 | 6.67 | 7.43 | 6.71 | 5.43 | 6.33 |
| **PL season** | 53.54 | 74.3 | 70.69 | 70.65 | 70.91 | 40.78 | 49.67 | 62.07 | 41.5 | 46.95 |
| **PL/Min season** | 1 | 1.07 | 1.09 | 1.04 | 1.06 | 0.98 | 0.75 | 0.98 | 0.91 | 0.83 |
| **HSR ABS (m) season** | 390.67 | 685.4 | 1116.93 | 421.36 | 518.98 | 125.55 | 162.11 | 359.52 | 228.75 | 215.79 |
| **HSR Rel (m) season** | 28.5 | 136.44 | 9.76 | 0 | 9.63 | 0 | 0 | 106.29 | 12.22 | 5.94 |
| **Season distance** | 3654.40 | 4576.58 | 4429.20 | 4226.62 | 4799.87 | 2847.20 | 3360.57 | 4363.63 | 2776.51 | 3324.22 |
| **Distance/Min season** | 67.35 | 66.07 | 68.35 | 62.14 | 71.43 | 68.53 | 50.586 | 69.20 | 61.05 | 59.03 |
| **ACC+2 (n) season** | 111.6 | 137625 | 164.77 | 200.11 | 244 | 86.5 | 143.66 | 164.11 | 91375 | 138875 |
| **DEC+2 (n) season** | 105 | 131625 | 148.55 | 187.44 | 232.33 | 81 | 131.66 | 152.77 | 82625 | 131625 |
| **ACC+2/Min (n) season** | 2.11 | 1.99 | 2.51 | 2.97 | 3.56 | 2.16 | 2.17 | 2.62 | 2.01 | 2.49 |
| **DEC+2/Min (n) season** | 1.980 | 1.91 | 2.26 | 2.78 | 3.35 | 2.003 | 1.99 | 2.44 | 1.81 | 2.34 |
| **ACC+2 (m) season** | 498.15 | 623.37 | 850.35 | 745.22 | 846.06 | 310.85 | 464.31 | 632.49 | 391.88 | 456.27 |
| **DEC+2 (m) season** | 391.64 | 468.95 | 649.28 | 598.79 | 691.77 | 238.035 | 345.44 | 523.84 | 291.04 | 359.98 |
| **ACC+2/Min (m) season** | 9.18 | 9.02 | 12.92 | 11.04 | 12.42 | 7.63 | 6.98 | 10.045 | 8.46 | 8.08 |
| **DEC+2/Min (m) season** | 7.22 | 6.75 | 9.84 | 8.86 | 10.02 | 5.83 | 5.18 | 8.37 | 6.32 | 6.30 |
| **Max PL season** | 75.77 | 84.62 | 96.8 | 92.69 | 91.02 | 49.85 | 59.13 | 76.56 | 58.27 | 76.72 |
