# Supplementary material for: Unraveling athletic performance: Transcriptomics and external load monitoring in handball competition
Source: PLoS One. 2024 Mar 11;19(3):e0299556. doi: 10.1371/journal.pone.0299556 (PMC10927131; doi:10.1371/journal.pone.0299556)
Supplement: S3 Table — (DOCX) [file pone.0299556.s003.docx]

**Table S3:** Correlation values between internal and external load match variables at baseline levels (Time 1).

| **EPTS variables** | **Pathways** | **Correlation value** | **Adjusted p-value** |
| --- | --- | --- | --- |
| **DEC+2 (n) TRANSC** | Fatty acid biosynthesis | 0.976 | 0.004 |
| **DEC+2/MIN (n) TRANSC** | Fatty acid biosynthesis | 0.976 | 0.004 |
| **ACC+2 (n) TRANS** | Amino sugar and nucleotide sugar metabolism | 0.961 | 0.014 |
| **ACC+2/MIN (n) TRANS** | Amino sugar and nucleotide sugar metabolism | 0.961 | 0.014 |
| **DEC+2 (n) TRANSC** | Amino sugar and nucleotide sugar metabolism | 0.949 | 0.023 |
| **DEC+2/MIN (n) TRANSC** | Amino sugar and nucleotide sugar metabolism | 0.949 | 0.023 |
| **ACC+2 (n) TRANS** | Fatty acid biosynthesis | 0.94 | 0.035 |
| **ACC+2/MIN (n) TRANS** | Fatty acid biosynthesis | 0.94 | 0.035 |
| **ACC+2 (n) TRANS** | TNF signaling pathway | 0.922 | 0.047 |
| **ACC+2/MIN (n) TRANS** | TNF signaling pathway | 0.922 | 0.047 |
