# Supplementary material for: Unraveling athletic performance: Transcriptomics and external load monitoring in handball competition
Source: PLoS One. 2024 Mar 11;19(3):e0299556. doi: 10.1371/journal.pone.0299556 (PMC10927131; doi:10.1371/journal.pone.0299556)
Supplement: S4 Table — (DOCX) [file pone.0299556.s004.docx]

**Table S4:** Correlation values between internal and external load variables after match (Time 2).

| **EPTS variables** | **Pathways** | **Correlation value** | **Adjusted p-value** |
| --- | --- | --- | --- |
| **PL/Min Transcriptoma** | Proteasome | 0.913 | 0.005 |
| **PL Transcriptoma** | Proteasome | 0.893 | 0.008 |
| **HSR ABS (m) TRAN** | Ribosome biogenesis in eukaryotes | 0.881 | 0.01 |
| **ACC+2 (m) TRANS** | Histidine metabolism | 0.873 | 0.011 |
| **ACC+2/Min (m) TRANS** | Histidine metabolism | 0.873 | 0.011 |
| **ACC+2 (m) TRANS** | Cardiac muscle contraction | 0.873 | 0.011 |
| **ACC+2/Min (m) TRANS** | Cardiac muscle contraction | 0.873 | 0.011 |
| **HSR ABS (m) TRAN** | Cardiac muscle contraction | 0.837 | 0.019 |
| **ACC+2 (m) TRANS** | SNARE interactions in vesicular transport | 0.827 | 0.021 |
| **ACC+2/Min (m) TRANS** | SNARE interactions in vesicular transport | 0.827 | 0.021 |
| **ACC+2 (n) TRANS** | ECM-receptor interaction | 0.828 | 0.021 |
| **ACC+2/MIN (n) TRANS** | ECM-receptor interaction | 0.828 | 0.021 |
| **HSR ABS (m) TRAN** | IL-17 signaling pathway | 0.824 | 0.022 |
| **HSR ABS (m) TRAN** | Basal transcription factors | 0.81 | 0.026 |
| **ACC+2 (m) TRANS** | Oxidative phosphorylation | 0.807 | 0.027 |
| **ACC+2/Min (m) TRANS** | Oxidative phosphorylation | 0.807 | 0.027 |
| **ACC+2 (m) TRANS** | Primary bile acid biosynthesis | 0.805 | 0.028 |
| **ACC+2/Min (m) TRANS** | Primary bile acid biosynthesis | 0.805 | 0.028 |
| **ACC+2 (n) TRANS** | Valine, leucine and isoleucine biosynthesis | 0.803 | 0.029 |
| **ACC+2/MIN (n) TRANS** | Valine, leucine and isoleucine biosynthesis | 0.803 | 0.029 |
| **HSR ABS (m) TRAN** | Cysteine and methionine metabolism | 0.799 | 0.03 |
| **ACC+2 (m) TRANS** | IL-17 signaling pathway | 0.799 | 0.03 |
| **ACC+2/Min (m) TRANS** | IL-17 signaling pathway | 0.799 | 0.03 |
| **ACC+2 (m) TRANS** | Gap junction | 0.795 | 0.031 |
| **ACC+2/Min (m) TRANS** | Gap junction | 0.795 | 0.031 |
| **DEC+2 (m) TRANS** | Cysteine and methionine metabolism | 0.789 | 0.033 |
| **ACC+2 (m) TRANS** | Renin secretion | 0.791 | 0.033 |
| **ACC+2/Min (m) TRANS** | Renin secretion | 0.791 | 0.033 |
| **DEC+2/Min (m) TRANS** | Cysteine and methionine metabolism | 0.789 | 0.034 |
| **DEC+2 (n) TRANSC** | Tyrosine metabolism | 0.787 | 0.034 |
| **DEC+2/MIN (n) TRANSC** | Tyrosine metabolism | 0.787 | 0.034 |
| **ACC+2 (n) TRANS** | Renin-angiotensin system | 0.782 | 0.036 |
| **ACC+2/MIN (n) TRANS** | Renin-angiotensin system | 0.782 | 0.036 |
| **ACC+2 (n) TRANS** | Cysteine and methionine metabolism | 0.779 | 0.038 |
| **ACC+2/MIN (n) TRANS** | Cysteine and methionine metabolism | 0.779 | 0.038 |
| **ACC+2 (n) TRANS** | Tyrosine metabolism | 0.775 | 0.039 |
| **ACC+2/MIN (n) TRANS** | Tyrosine metabolism | 0.775 | 0.039 |
| **DEC+2 (n) TRANSC** | Citrate cycle (TCA cycle) | 0.772 | 0.04 |
| **DEC+2/MIN (n) TRANSC** | Citrate cycle (TCA cycle) | 0.772 | 0.04 |
| **HSR ABS (m) TRAN** | Histidine metabolism | 0.772 | 0.04 |
| **DEC+2 (n) TRANSC** | ECM-receptor interaction | 0.774 | 0.04 |
| **DEC+2/MIN (n) TRANSC** | ECM-receptor interaction | 0.774 | 0.04 |
| **ACC+2 (n) TRANS** | Citrate cycle (TCA cycle) | 0.769 | 0.042 |
| **ACC+2/MIN (n) TRANS** | Citrate cycle (TCA cycle) | 0.769 | 0.042 |
| **ACC+2 (m) TRANS** | Phagosome | 0.768 | 0.042 |
| **ACC+2/Min (m) TRANS** | Phagosome | 0.767 | 0.042 |
| **ACC+2 (n) TRANS** | Ribosome | 0.763 | 0.044 |
| **ACC+2/MIN (n) TRANS** | Ribosome | 0.763 | 0.044 |
| **DEC+2 (m) TRANS** | IL-17 signaling pathway | 0.763 | 0.044 |
| **DEC+2/Min (m) TRANS** | IL-17 signaling pathway | 0.763 | 0.044 |
| **DEC+2 (n) TRANSC** | Valine, leucine and isoleucine biosynthesis | 0.762 | 0.045 |
| **DEC+2/MIN (n) TRANSC** | Valine, leucine and isoleucine biosynthesis | 0.761 | 0.045 |
| **ACC+2 (m) TRANS** | Ascorbate and aldarate metabolism | 0.756 | 0.047 |
| **ACC+2/Min (m) TRANS** | Ascorbate and aldarate metabolism | 0.756 | 0.047 |
| **HSR ABS (m) TRAN** | Gap junction | 0.754 | 0.049 |
