# Supplementary material for: Unraveling athletic performance: Transcriptomics and external load monitoring in handball competition
Source: PLoS One. 2024 Mar 11;19(3):e0299556. doi: 10.1371/journal.pone.0299556 (PMC10927131; doi:10.1371/journal.pone.0299556)
Supplement: S5 Table — (DOCX) [file pone.0299556.s005.docx]

**Table S5:** Correlation values between internal and external load match variables 24 hours after finishing the match (Time 3).

| **EPTS variables** | **Pathways** | **Correlation value** | **Adjusted p-value** |
| --- | --- | --- | --- |
| **DEC+2 (n) TRANSC** | Valine, leucine and isoleucine biosynthesis | 0.845 | 0.018 |
| **DEC+2/MIN (n) TRANSC** | Valine, leucine and isoleucine biosynthesis | 0.845 | 0.018 |
| **ACC+2 (n) TRANS** | Valine, leucine and isoleucine biosynthesis | 0.843 | 0.019 |
| **ACC+2/MIN (n) TRANS** | Valine, leucine and isoleucine biosynthesis | 0.843 | 0.019 |
| **ACC+2 (n) TRANS** | Base excision repair | 0.836 | 0.02 |
| **ACC+2/MIN (n) TRANS** | Base excision repair | 0.836 | 0.02 |
| **DEC+2 (m) TRANS** | One carbon pool by folate | 0.825 | 0.023 |
| **DEC+2/Min (m) TRANS** | One carbon pool by folate | 0.825 | 0.023 |
| **ACC+2 (n) TRANS** | Arginine and proline metabolism | 0.817 | 0.025 |
| **ACC+2/MIN (n) TRANS** | Arginine and proline metabolism | 0.817 | 0.025 |
| **DEC+2 (n) TRANSC** | TGF-beta signaling pathway | 0.816 | 0.025 |
| **DEC+2/MIN (n) TRANSC** | TGF-beta signaling pathway | 0.816 | 0.025 |
| **DEC+2 (n) TRANSC** | GnRH secretion | 0.812 | 0.026 |
| **DEC+2/MIN (n) TRANSC** | GnRH secretion | 0.812 | 0.026 |
| **DEC+2 (n) TRANSC** | Base excision repair | 0.809 | 0.027 |
| **DEC+2/MIN (n) TRANSC** | Base excision repair | 0.808 | 0.027 |
| **DEC+2 (n) TRANSC** | Hippo signaling pathway | 0.811 | 0.027 |
| **DEC+2/MIN (n) TRANSC** | Hippo signaling pathway | 0.811 | 0.027 |
| **ACC+2 (n) TRANS** | p53 signaling pathway | 0.807 | 0.028 |
| **ACC+2/MIN (n) TRANS** | p53 signaling pathway | 0.807 | 0.028 |
| **ACC+2 (n) TRANS** | TGF-beta signaling pathway | 0.797 | 0.031 |
| **ACC+2/MIN (n) TRANS** | TGF-beta signaling pathway | 0.797 | 0.031 |
| **PL/Min Transcriptoma** | Glycine,serine and threonine metabolism | 0.789 | 0.034 |
| **ACC+2 (n) TRANS** | Hippo signaling pathway | 0.787 | 0.034 |
| **ACC+2/MIN (n) TRANS** | Hippo signaling pathway | 0.787 | 0.034 |
| **ACC+2 (n) TRANS** | GnRH secretion | 0.783 | 0.035 |
| **ACC+2/MIN (n) TRANS** | GnRH secretion | 0.783 | 0.035 |
| **DEC+2 (n) TRANSC** | Arginine and proline metabolism | 0.782 | 0.036 |
| **DEC+2/MIN (n) TRANSC** | Arginine and proline metabolism | 0.782 | 0.036 |
| **PL Transcriptoma** | Glycine,serine and threonine metabolism | 0.777 | 0.038 |
| **DEC+2 (n) TRANSC** | p53 signaling pathway | 0.777 | 0.038 |
| **DEC+2/MIN (n) TRANSC** | p53 signaling pathway | 0.777 | 0.038 |
| **ACC+2 (n) TRANS** | Longevity regulating pathway | 0.771 | 0.04 |
| **DEC+2 (n) TRANSC** | Longevity regulating pathway | 0.77 | 0.04 |
| **ACC+2/MIN (n) TRANS** | Longevity regulating pathway | 0.771 | 0.04 |
| **DEC+2/MIN (n) TRANSC** | Longevity regulating pathway | 0.77 | 0.04 |
| **ACC+2 (n) TRANS** | AMPK signaling pathway | 0.758 | 0.045 |
| **DEC+2 (n) TRANSC** | AMPK signaling pathway | 0.758 | 0.045 |
| **ACC+2/MIN (n) TRANS** | AMPK signaling pathway | 0.758 | 0.045 |
| **DEC+2/MIN (n) TRANSC** | AMPK signaling pathway | 0.758 | 0.045 |
