# Supplementary material for: Unraveling athletic performance: Transcriptomics and external load monitoring in handball competition
Source: PLoS One. 2024 Mar 11;19(3):e0299556. doi: 10.1371/journal.pone.0299556 (PMC10927131; doi:10.1371/journal.pone.0299556)
Supplement: S6 Table — (DOCX) [file pone.0299556.s006.docx]

**Table S6**: Correlation values between internal and external load season average variables just after finishing the match (Time 2).

| **EPTS variables** | **Pathways** | **Correlation value** | **Adjusted p-value** |
| --- | --- | --- | --- |
| **HSR ABS (m) Temp** | Histidine metabolism | 0.928 | 0.004 |
| **HSR ABS (m) Temp** | Proteasome | 0.885 | 0.009 |
| **HSR ABS (m) Temp** | Cardiac muscle contraction | 0.87 | 0.012 |
| **HSR ABS (m) Temp** | Gap junction | 0.846 | 0.017 |
| **HSR ABS (m) Temp** | Ascorbate and aldarate metabolism | 0.839 | 0.018 |
| **DEC+2/Min (m) TEMPORADA** | Hippo signaling pathway | 0.839 | 0.018 |
| **HSR ABS (m) Temp** | Phagosome | 0.835 | 0.019 |
| **ACC+2/Min (m) TEMPORADA** | Hippo signaling pathway | 0.834 | 0.02 |
| **DEC+2/Min (m) TEMPORADA** | Other types of O-glycan biosynthesis | 0.821 | 0.023 |
| **DEC+2/Min (m) TEMPORADA** | Biosynthesis of unsaturated fatty acids | 0.819 | 0.024 |
| **ACC+2/Min (m) TEMPORADA** | Proteasome | 0.812 | 0.026 |
| **ACC+2/MIN (n) TEMPORADA** | Phosphonate and phosphinate metabolism | 0.81 | 0.027 |
| **DEC+2/MIN (n) TEMPORADA** | Phosphonate and phosphinate metabolism | 0.806 | 0.028 |
| **ACC+2/Min (m) TEMPORADA** | Biosynthesis of unsaturated fatty acids | 0.8 | 0.03 |
| **ACC+2/Min (m) TEMPORADA** | Other types of O-glycan biosynthesis | 0.79 | 0.034 |
| **HSR ABS (m) Temp** | Ubiquinone and other terpenoid-quinone biosynthesis | 0.786 | 0.035 |
| **HSR ABS (m) Temp** | Cell cycle | 0.784 | 0.036 |
| **ACC+2 (n) TEMPORADA** | Phosphonate and phosphinate metabolism | 0.782 | 0.037 |
| **DEC+2 (n) TEMPORADA** | Phosphonate and phosphinate metabolism | 0.782 | 0.037 |
| **ACC+2 (m) TEMPORADA** | Proteasome | 0.779 | 0.038 |
| **DEC+2 (m) TEMPORADA** | Other types of O-glycan biosynthesis | 0.774 | 0.04 |
| **DEC+2 (m) TEMPORADA** | Biosynthesis of unsaturated fatty acids | 0.763 | 0.045 |
| **DEC+2/Min (m) TEMPORADA** | Long-term potentiation | 0.761 | 0.046 |
| **HSR ABS (m) Temp** | Oxidative phosphorylation | 0.759 | 0.047 |
| **ACC+2/Min (m) TEMPORADA** | Ubiquitin mediated proteolysis | 0.759 | 0.047 |
| **DEC+2/Min (m) TEMPORADA** | Ubiquitin mediated proteolysis | 0.758 | 0.047 |
| **DEC+2/Min (m) TEMPORADA** | Hedgehog signaling pathway | 0.758 | 0.047 |
