# Supplementary material for: Unraveling athletic performance: Transcriptomics and external load monitoring in handball competition
Source: PLoS One. 2024 Mar 11;19(3):e0299556. doi: 10.1371/journal.pone.0299556 (PMC10927131; doi:10.1371/journal.pone.0299556)
Supplement: S7 Table — (DOCX) [file pone.0299556.s007.docx]

**Table S7:** Correlation values between internal and external load season average variables 24 hours after finishing the match (Time 3).

| **EPTS variables** | **Pathways** | **Correlation value** | **Adjusted p-value** |
| --- | --- | --- | --- |
| **DEC+2 (n) TEMPORADA** | Insulin secretion | 0.928 | 0.004 |
| **HSR ABS (m) Temp** | Glycine, serine and threonine metabolism | 0.839 | 0.018 |
| **Max PL Temporada** | Adipocytokine signaling pathway | 0.81 | 0.027 |
| **Max PL Temporada** | Arachidonic acid metabolism | 0.786 | 0.035 |
| **ACC+2/MIN (n) TEMPORADA** | Insulin secretion | 0.782 | 0.037 |
| **DEC+2/MIN (n) TEMPORADA** | Insulin secretion | 0.782 | 0.037 |
| **ACC+2 (n) TEMPORADA** | Insulin secretion | 0.759 | 0.047 |
